# Development of a Natural Language Processing Tool to Extract Radiation Treatment Sites

**DOI:** 10.7759/cureus.6010

**Published:** 2019-10-28

**Authors:** Gary Walker, Ergin Soysal, Hua Xu

**Affiliations:** 1 Radiation Oncology, Banner MD Anderson Cancer Center, Gilbert, USA; 2 School of Biomedical Informatics, The University of Texas Medical School at Houston, Houston, USA

**Keywords:** radiation therapy, natural language processing, treatment site

## Abstract

Currently, radiation oncology-specific electronic medical records (EMRs) allow providers to input the radiation treatment site using free text. The purpose of this study is to develop a natural language processing (NLP) tool to extract encoded data from radiation treatment sites in an EMR.

Treatment sites were extracted from all patients who completed treatment in our department from April 1, 2011, to April 30, 2013. A system was designed to extract the Unified Medical Language System (UMLS) concept codes using a sample of 11,018 unique site names from 31118 radiation therapy (RT) sites. Among those, 5500 unique site name strings that constitute approximately half of the sample were spared as a test set to evaluate the final system. A dictionary and calculated n-gram statistics using UMLS concepts from related semantic types were combined with manually encoded data.

There was an average of 2.2 sites per patient. Prior to extraction, the 20 most common unique treatment sites were used 4215 times (38.3%). The most common treatment site was whole brain RT, which was entered using 27 distinct terms for a total of 1063 times. The customized NLP solution displayed great gains as compared to other systems, with a recall of 0.99 and a precision of 0.99.

A customized NLP tool was extracting encoded data from radiation treatment sites in an EMR with great accuracy. This can be integrated into a repository of demographic, genomic, treatment, and outcome data to advance personalized oncologic care.

## Introduction

A National Radiation Oncology Registry (NROR) has been created through a collaboration between the Radiation Oncology Institute (ROI) and the American Society for Radiation Oncology (ASTRO) to develop a national database that will be used to study the clinical outcomes and patterns of care. Inherent in this effort is the need for automated tools to extract clinical information from radiation oncology electronic medical records (EMRs). Currently, radiation oncology-specific electronic medical records allow providers to input the treatment site using free text, leading to a glut of potential options. This paradigm creates great challenges in answering research questions that depend on assembling a cohort of patients who received similar treatments.

Previous studies have demonstrated the feasibility of extracting meaningful clinical data using natural language processing (NLP) tools from diagnoses [1], problem lists [2], pathology reports [3-4], and radiology reports [5-8]. These tools are not designed to handle the complexities of radiation therapy (RT) site names, which include many abbreviations specific to our field. The purpose of this study is to develop an NLP tool to extract encoded data from radiation treatment sites in an EMR.

## Technical report

For this analysis, information was obtained from the RT delivery (record and verify) electronic medical record (MOSAIQ®, Elekta Care Management, Stockholm), which allows manual, free-text input of the desired treatment site. Treatment sites were extracted from all patients who completed treatment in our department from April 1, 2011, to April 30, 2013. A separate treatment site was entered for each radiation field. For example, a breast RT treatment might consist of three treatment sites in the RT prescription: 1) left breast field, 2) internal mammary chain field, and 3) supraclavicular field. In general, at that time, our department did not have a standardized nomenclature for labeling treatment sites. In addition, we practiced in a large department with multiple physicians per clinical service, leading to a large heterogeneity in the labeling of treatment sites. The study was deemed to be exempt from review by the Institutional Review Board as a quality improvement project.

A system was designed to extract the Unified Medical Language System (UMLS) concept codes using a sample of 11,018 unique site names from 31118 RT sites. Among those, 5500 unique site name strings that constituted approximately half of the sample were spared as a test set to evaluate the final system, and the remaining site name entries were used for system development.

As an initial requirement, we developed a dictionary and calculated n-gram statistics using UMLS concepts from related semantic types like Body Part, Organ, or Organ Component (T023), Body Location or Region (T029), Body Space or Junction (T030), or Spatial Concept (T082 ) that represented topographical entities within the body or other concepts. Although the majority of the concepts are covered by this UMLS subset, there were still uncovered concepts specific to the radiation therapy domain. These concepts and common abbreviations from the development set were manually extracted to create a supplement terminology to overcome the shortcomings of UMLS.

The system first processed a site name into tokens and then each token was matched against the above dictionary. If the token was not in the dictionary, the English dictionary was used to identify the word. Because of arbitrary abbreviations ( for example, supraclavicular can be abbreviated as SCV, SCL, S/C, SC, S Clav, Sc V, Sclav, or SCLV), the system frequently returned an unknown token. In these cases, if there was no match, a list of possible candidates was obtained by preceding or following the token from the application terminology based on bigrams. For each candidate, the Levenshtein distance was calculated, and the closest word having the highest probability was chosen as the correct one. After this word identification step, the site name was processed to build terms, in comparison to the UMLS concept subset.

For the evaluation of the system, the application was run on the spared site names for test purposes. The output was compared to a review of site names by a radiation oncologist as the gold standard.

The mean number of times a unique treatment site was used among all patients was 2.82 (range 1-450). There was an average of 2.2 sites per patient. Prior to extraction, the 20 most common unique treatment sites were used 4215 times (38.3%) (Table 1).

**Table 1:** The 20 most frequent treatment sites with natural language processing extracted clinical concepts SCV: supraclavicular

Site Name	Frequency	Concept Identified 1	Concept Identified 2	Concept Identified 3	Concept Identified 4
Pelvis	450	pelvis [C0030797,C0559769,C0030786,C1279864:T023,T029,T030]			
SCV	428	supraclavicular region [C0446461:T029]			
Whole Brain	298	whole [C0444667:T081]	brain [C0006104,C1269537,C1882598,C1273723:T023,T029]		
whole brain	266	whole [C0444667:T081]	Brain [C0006104,C1269537,C1882598,C1273723:T023,T029]		
Tumor Bed Boost	262	tumor [C0027651,C3273930,C1578706:T191,T170,T033]	bed [C1547114:T082]	Boost [C1511253:T169]	
Left Breast	260	Left Breast [C0222601:T023]			
Right Chest Wall	241	right [C0205090:T082]	Chest wall [C0205076:T023]		
Right Breast	239	Right Breast [C0222600:T023]			
Left Chest Wall	206	Left [C0205091:T082]	Chest wall [C0205076:T023]		
pelvis	198	pelvis [C0030797,C0559769,C0030786,C1279864:T023,T029,T030]			
Boost	194	Boost [C1511253:T169]			
Whole brain	189	whole [C0444667:T081]	Brain [C0006104,C1269537,C1882598,C1273723:T023,T029]		
Prostate/Prox SV	159	Prostate [C0033572,C1278980,C1882832:T023]	/ [PUNC:T000]	Proximal [C0205107:T082] (222.0)	Seminal Vesicle [C1278984:T023]
boost	148	Boost [C1511253:T169]			
SCV-MLB	147	supraclavicular region [C0446461:T029]	midline block [:T169]		
SCV MLB	139	supraclavicular region [C0446461:T029]	midline block [:T169]		
Right breast	134	Right Breast [C0222600:T023]			
Prostate	129	Prostate [C0033572,C1278980,C1882832:T023]			
WBRT	128	whole-brain radiotherapy [C1520143:T061]			

The customized NLP solution displayed great gains as compared to other systems, with a recall of 0.99 and a precision of 0.99 (Table 2). 

**Table 2:** Recall and precision for the customized natural language processing system, as compared to two common NLP tools (MetaMap and cTAKES) NLP: natural language processing; cTAKES: clinical text analysis and knowledge extraction system

Application	Recall TP/(TP+FN)	Precision TP/(TP+FP)
MetaMap	0.51	0.75
cTAKES	0.46	0.85
Customized NLP System	0.99	0.99

The most common treatment site was whole brain RT, which was entered using 27 distinct terms for a total of 1063 times (Table 3).

**Table 3:** Variations in describing treatment site for whole-brain radiation therapy PCI: percutaneous coronary intervention; WBRT: whole-brain radiation therapy; WBXRT: whole-brain radiotherapy

Name	Frequency
Whole Brain	298
whole brain	266
Whole brain	189
WBRT	128
Brain	113
brain	21
wbxrt	8
WHOLE BRAIN	6
Whole brain 2	3
whole brain PCI	3
whole Brain	3
Whole Brain #2	3
Whole Brain PCI	3
Whole Brain Retreat	2
WHole Brain	2
whole brain 1	2
Whole Brain RT	2
WBRT (PCI)	2
whole brain-2	1
Whole Brain photons	1
PCI, whole brain	1
Whole brain/eyes/BOS	1
Whole Brain Proton	1
whole brain modulate	1
whole brain 2	1
Whole brain Primary	1
Whole Brain-PCI	1

## Discussion

This study analyzed a cohort of RT treatment sites and developed a customized NLP system that can extract structured data with very high recall and precision compared to non-customized tools. A method to extract structured data from RT treatment sites has not been described in the literature.

This tool can be incorporated into an institutional data warehouse as a repository of integrated genomic sequencing data, treatment details, and outcome data. This data will allow us to make better treatment decisions and predict individual patients’ risk of acute and long-term toxicity due to oncologic therapy and thus further personalize their care.

Currently, the majority of cancer registries and departmental databases rely on manual coding to determine receipt of RT. With the widespread adoption of EMRs, automated coding will become increasingly important. NLP has the potential to facilitate this reporting in a structured, meaningful way. This tool could automate the reporting of treatment fields to improve the quality and accuracy of retrospective and prospective research, thus improving their meaningful use. In addition, similar tools can be developed for other radiation oncology applications such as extracting coded data from dose-volume histograms (DVHs).

This study has certain limitations, which need to be addressed. While these results are compelling, they only apply to our unique clinical workflow. Some institutions may have a more structured process for entering treatment sites, leading to differing results with a similar tool.

## Conclusions

In summary, we developed an NLP tool to extract encoded data from radiation treatment sites in an EMR. This can be integrated into a repository of demographic, genomic, treatment, and outcome data to advance personalized oncologic care.

## References

[1] Zeng QT, Goryachev S, Weiss S, Sordo M, Murphy SN, Lazarus R (2006). Extracting principal diagnosis, co-morbidity and smoking status for asthma research: evaluation of a natural language processing system. BMC Med Inform Decis Mak.

[2] Meystre S, Haug PJ (2005). Automation of a problem list using natural language processing. BMC Med Inform Decis Mak.

[3] Xu H, Friedman C (2003). Facilitating research in pathology using natural language processing. AMIA Annu Symp Proc.

[4] Imler TD, Morea J, Kahi C, Imperiale TF (2013). Natural language processing accurately categorizes findings from colonoscopy and pathology reports. Clin Gastroenterol Hepatol.

[5] Soysal E, Cicekli I, Baykal N (2010). Design and evaluation of an ontology based information extraction system for radiological reports. Comput Biol Med.

[6] Lacson R, Khorasani R (2011). Practical examples of natural language processing in radiology. J Am Coll Radiol.

[7] Friedman C, Alderson PO, Austin JH, Cimino JJ, Johnson SB (1994). A general natural-language text processor for clinical radiology. J Am Med Inform Assoc.

[8] Hripcsak G, Austin JH, Alderson PO, Friedman C (2002). Use of natural language processing to translate clinical information from a database of 889,921 chest radiographic reports. Radiology.

